# Longitudinal measurement invariance and psychometric properties of the Patient Health Questionnaire-Four in China

**DOI:** 10.1186/s12888-024-05873-2

**Published:** 2024-07-22

**Authors:** Runtang Meng, Chen Jiang, Joseph M. Dzierzewski, Yihong Zhu, Meng Wang, Nongnong Yang, Xiaoxue Liu, Lina Guo, Yufan Ping, Caojie Zhou, Jiale Xu, Wenjing Zou, Xiaowen Wang, Liping Lu, Haiyan Ma, Yi Luo, Karen Spruyt

**Affiliations:** 1 School of Public Health, Hangzhou Normal University, Hangzhou, 311121 Zhejiang China; 2 Engineering Research Center of Mobile Health Management System, Ministry of Education, Hangzhou, Zhejiang China; 3 National Sleep Foundation, Washington, DC USA; 4 School of Clinical Medicine, Hangzhou Normal University, Hangzhou, Zhejiang China; 5 Ophthalmology Center, Ningbo Yinzhou No.2 Hospital, Ningbo, Zhejiang China; 6 Global Health Research Division, Public Health Research Center and Department of Public Health and Preventive Medicine, Wuxi School of Medicine, Jiangnan University, Wuxi, Jiangsu China; 7 Department of Neurology, The First Affiliated Hospital of Zhengzhou University, Zhengzhou, Henan China; 8 School of Nursing, Ningbo College of Health Sciences, Ningbo, Zhejiang China; 9 Université Paris Cité, NeuroDiderot, INSERM, Paris, France

**Keywords:** Patient Health Questionnaire-4, Confirmatory factor analysis, Longitudinal measurement invariance, Psychometric properties, Healthcare students

## Abstract

**Background:**

Depression and anxiety symptoms among medical students are often a concern. The Patient Health Questionnaire-Four (PHQ-4), an important tool for depression and anxiety screening, is commonly used and easy to administer. This study aimed to assess and update the longitudinal measurement invariance and psychometric properties of the simplified Chinese version.

**Methods:**

A three-wave longitudinal survey was conducted among healthcare students using the PHQ-4. Structural validity was based on one-factor, two-factor, and second-order factor models, construct validity was based on the Self-Rated Health Questionnaire (SRHQ), Sleep Quality Questionnaire (SQQ), and Rosenberg Self-Esteem Scale (RSES), and longitudinal measurement invariance (LMI), internal consistency, and test–retest reliability were based on structural consistency across three time points.

**Results:**

The results of the confirmatory factor analysis indicated that two-factor model was the best fit, and LMI was supported at three time points. Inter-factor, factor-total, and construct validity correlations of the PHQ-4 were acceptable. Additionally, Cronbach’s alpha, McDonald’s omega, and the intraclass correlation coefficient demonstrated acceptable/moderate to excellent reliability of the PHQ-4.

**Conclusions:**

This study adds new longitudinal evidence that the Chinese version of the PHQ-4 has promising LMI and psychometric properties. Such data lends confidence to the routine and the expanded use of the PHQ-4 for routine screening of depression and anxiety in Chinese healthcare students.

**Supplementary Information:**

The online version contains supplementary material available at 10.1186/s12888-024-05873-2.

## Background

The most common mental disorders in both clinical patients and the general population are depression and anxiety, which often co-occur [1–5]. Depression and anxiety account for more than half of mental disorders worldwide [6, 7]. Well known is that the co-occurrence of depression and anxiety is associated with significant disability and symptom severity, such as low back pain, poor social functioning, and multiple sclerosis [8–12].

Depression and anxiety usually first appear in adolescence, some of the symptoms may be acute; however, depression and anxiety can both also be chronic in nature, resulting in a huge public health burden [13–15]. Substantial existing evidence indicates a high prevalence of depression and anxiety among healthcare students, with overall levels of psychological distress consistently higher than the general population and peers [16–22]. The negative impact of psychological distress is far-reaching, which may adversely affect academic performance, decrease empathy, and elevate burnout in healthcare careers [23–25].

As such, it is widely accepted that depression and anxiety should be routinely assessed and, if present, first-line treatment should be applied to improve outcomes [26–28]. The 4-item Patient Health Questionnaire (PHQ-4) has been widely used as a screener due to its ultra-short nature [26, 27]. Consisting of the first two items from the Patient Health Questionnaire-9 (PHQ-9) to assess depression [27, 29] and the first two items from the Generalized Anxiety Disorder-7 (GAD-7) to measure anxiety [30], the PHQ-4 (i.e., PHQ-2 plus GAD-2) has been translated into several languages including Spanish, German, Greek, and Korean [26, 31–35]. Moreover, the PHQ-4 has been validated in a variety of populations (e.g., patients, students, pregnant women, athletes, and adolescents) [26, 31–43]. Whether in a distinctive cultural background or population setting, the PHQ-4 has demonstrated a stable two-factor structure (comparative fit index [CFI] = 0.990–1.000, Tucker-Lewis index [TLI] = 0.980–1.000, root mean square error of approximation [RMSEA] = 0.011–0.080), valid construct validity (adequate convergent and discriminant validity), and good reliability (Cronbach’s alpha = 0.720–0.880, McDonald’s omega = 0.850–0.880) [32, 35–47]. However, there is little evidence that the Chinese cultural adaptation examining whether the PHQ-4 has retained a stable two-factor structure consistent with its design [35]. Only one study could be found that applied the traditional Chinese version of the PHQ-4 among Hong Kong young adults [48]. The measurement properties of the simplified Chinese version are therefore worth discovering [35, 48].

Most importantly, it remains to be seen whether the PHQ-4 displays longitudinal measurement invariance (LMI) [39]. Of the existing evidence, only the Greek version of the PHQ-4 examined repeated surveys to assess its test–retest reliability, yet the LMI was not assessed [34]. As an ultra-short instrument to screen for depression and anxiety, and track changes in these symptoms, LMI is essential to demonstrate that the construct has the same meaning across repeated assessments [27, 34, 49]. Given the specific nature of depression and anxiety, which can occur acutely (e.g., 7 to 21 days) and chronically (e.g., weeks to years), both intervals of short-term and relatively long are worth exploring [11, 50–54].

The purpose of the current study was to address the following questions: 1) is the factor structure of the simplified Chinese PHQ-4 stable as a two-factor model; and 2) would the simplified Chinese PHQ-4 demonstrate adequate LMI across both short- and long-term intervals? Longitudinal measurement invariance and adequate psychometric properties for the PHQ-4 would support continued and future routine and general screening with this tool [11, 26, 39, 51, 52]. Early identification and targeted prevention programs could help to prevent episodes of depression and anxiety in healthcare students [13, 55, 56].

## Methods

### Study design and participants

A three-wave longitudinal survey was conducted from December 2020 to April 2021 in Hangzhou, China. All healthcare students freely consented to answer the questionnaires. The study was approved by the Institutional Review Board of Hangzhou Normal University Division of Health Sciences, China. All procedures followed the relevant ethical tenets of the Declaration of Helsinki [57].

Healthcare students enrolled in medical courses were recruited based on the following inclusion criteria: 1) aged 17–24 years old as undergraduates; 2) not diagnosed with mental disorders; and 3) volunteered to participate in the survey. Excluded participants were primarily: 1) international exchange students who did not fully understand Chinese; and 2) on long-term leave (i.e., ≥ 3 months) for medical internship or suspension. Surveys were administered three times as baseline (T1), one-week follow-up (T2), and 15-week follow-up (T3) to allow for analyses across intervals that mimic real-world need [51–54, 58, 59].

A total of 637, 616, and 540 participants completed questionnaires at baseline, one-week follow-up, and 15-week follow-up timepoints, respectively. A total of 512 paper–pencil questionnaires were considered valid after matching. The final sample size met basic sample size considerations, which included the following: 1) the sample size should be at least 10 times the number of items in the scale; and 2) the sample size should be higher than 500 considering the estimated ratio of items to factors in the study is 2 [60, 61].

### Measures

#### Patient Health Questionnaire (Chinese Version)

The simplified Chinese version of the 4-item Patient Health Questionnaire (PHQ-4-SC, retrieved from: https://www.phqscreeners.com; accessed on 29 August 2019) was the focus of the present study [27, 30, 62]. The PHQ-4 consists of two core criteria for depression and another two for anxiety syndrome. Participants respond to the core prompt: “In the past 2 weeks, how often have the following problems bothered you?”, all items (e.g., “Feeling nervous, anxious or on edge”) are scored on a four-point scale marked with 0 (“not at all”), 1 (“several days”), 2 (“more than half the days”), and 3 (“nearly every day”). A higher score on the PHQ-4-SC indicates poorer mental health, with total scores ranging from 0 to 12.

#### Self-Rated Health Questionnaire (Chinese Version)

The Self-Rated Health Questionnaire (SRHQ) consists of two items measuring physical health and mental health, respectively [49, 63, 64]. Individuals self-report their perceived health status on a five-point Likert scale with response categories of “excellent = 1, good = 2, average = 3, poor = 4, and extremely poor = 5”. The higher the total score on the SRHQ (2 to 10), the better the self-perceived health. The Cronbach’s alpha of the SRHQ were 0.686, 0.672, and 0.750 at baseline, 1-week follow-up, and 15-week follow-up respectively for the current study.

#### Sleep Quality Questionnaire (Chinese Version)

The Chinese version of the Sleep Quality Questionnaire (SQQ-C) is a self-report scale that measures an individual’s sleep quality with two major subconstructs: daytime sleepiness and sleep disturbance [65, 66]. Using a five-point Likert scale ranging from “strongly agree” to “strongly disagree” (0 to 4), higher scores indicate poorer sleep quality. The SQQ has demonstrated adequate measurement properties in a multi-center study (CFI = 0.903–0.977, TLI = 0.872–0.969, RMSEA = 0.073–0.142; Cronbach’s alpha = 0.712–0.862, McDonald’s omega = 0.723–0.863) [67].

#### Rosenberg Self-Esteem Scale (Chinese Version)

The Chinese version of the Rosenberg Self-Esteem Scale (RSES-C), one of the most widely used self-esteem instruments in the world, has two core dimensions: 1) positively worded items are scored from 1 (strongly agree) to 4 (strongly disagree); and 2) negatively worded items are reversed scored, from 1 (strongly disagree) to 4 (strongly agree) [68]. After reversing the item scores, the total score ranges from 10 to 40 with higher scores representing higher self-esteem; and the RSES-C demonstrated sound measurement properties [69].

### Statistical analysis

All data were assembled in EpiData (version 3.1). R (version 4.2.1) and its compiler RStudio (version 2022.12.0) were used to perform the statistical analysis with the following packages: “*MVN*”, “*lavaan*”, “*semTools*”, and “*ufs*” [70–73]. Guided by the COnsensus-based Standards for the selection of health Measurement INstruments (COSMIN) methodology manual and taxonomy of measurement properties, we aim to assess the structural validity, construct validity, longitudinal measurement invariance, and internal consistency of the PHQ-4-SC [74–76].

### Structural validity

Confirmatory factor analysis (CFA) was first applied to determine whether the two-factor structure is consistent with the original design. The one-factor model (i.e., 4 items loaded on a general factor: psychological distress/functioning) and the two-order factor model (i.e., 2 items loaded on a depression factor and the other 2 items loaded on an anxiety factor) were selected as competing factor structures. An illustration can be found in Figure S1 of Supplementary Material. Weighted least squares mean- and variance-adjusted (WLSMV) estimation was used in all CFA analyses taking into account the ordinal nature of the item scores [77–79]. All of the listed competing structures of the PHQ-4-SC were examined by the CFI, TLI, and RMSEA [71, 80–83]. The goodness-of-fit (GOF) of the PHQ-4-SC was determined by thresholds (CFI ≥ 0.900, TLI ≥ 0.900, and RMSEA ≤ 0.080), the model could be considered the least suitable [83]. The model with the relatively best GOF performances was selected for all subsequent analyses.

### Longitudinal measurement invariance

Parameters were progressively constrained to test the LMI of the chosen structural model: configural, threshold, metric, scalar, and strict models (Supplementary Material in Table S1) [49]. The scaled GOF indices (CFI, TLI, and RMSEA) together with their changes (Δ) as absolute values were used to assess LMI: 1) CFI ≥ 0.900, TLI ≥ 0.900, and RMSEA ≤ 0.080 were the least required cut-offs; and 2) |ΔCFI|≤ 0.010, |ΔTLI|≤ 0.010, and |ΔRMSEA|≤ 0.015 were the least required cut-offs. Once two, one, or no GOF indices had Δs found to fall outside the cutoffs, the model judged to be unsupported (marked red), nearly supported (marked yellow), or supported (marked green), respectively [49]. The chi-squared statistic (*χ*^*2*^) and the chi-square change (Δ*χ*^*2*^) were also compared between the models as secondary indicators, as they are sensitive to the sample size.

### Construct validity

Guided by the COSMIN guidelines, we made the following hypotheses regarding construct validity [84, 85]:

1. The PHQ-4-SC would positively correlate (0.300–0.500) with the SRHQ, as both measure related constructs but the SRHQ tends to focus more on health conditions.
2. The PHQ-4-SC would positively correlate (0.300–0.500) with the SQQ, as both measure related constructs but the SQQ tends to focus more on sleep quality.
3. The PHQ-4-SC would positively correlate (0.300–0.500) with the RSES, as both measure related constructs but the RSES tends to focus more on self-esteem.

All of the three hypotheses (75%) had to be fulfilled for sufficient construct validity.

### Internal consistency and test–retest reliability

The ordinal forms of Cronbach’s alpha, McDonald’s omega, and their 95% confidential interval were calculated to assess the internal consistency of the measures [86, 87]. Internal consistency would be considered adequate if both the alpha and omega were greater than or equal to 0.700 [84, 88].

In terms of test–retest reliability, we calculated the intraclass correlation coefficient (ICC) to measure stability across timepoints. An ICC would be considered poor if it was less than 0.500, moderate if it was between 0.500 and 0.750, good if it was between 0.750 and 0.900, or excellent if it was greater than 0.900 [89–91]. The standard error of measurement (SEM) was also calculated as an additional indicator of test–retest reliability using the formula “standard deviation × sqrt (1-ICC)” [89].

## Results

### Sample characteristics

A total of 512 valid participants were included in this study. The mean age of the sample is 20.219 years, and 77.0% were female. The other demographic information and the total score of the PHQ-4-SC are summarized in the Supplementary Material, Table S2.

### Structural validity

**Fig. 1:**
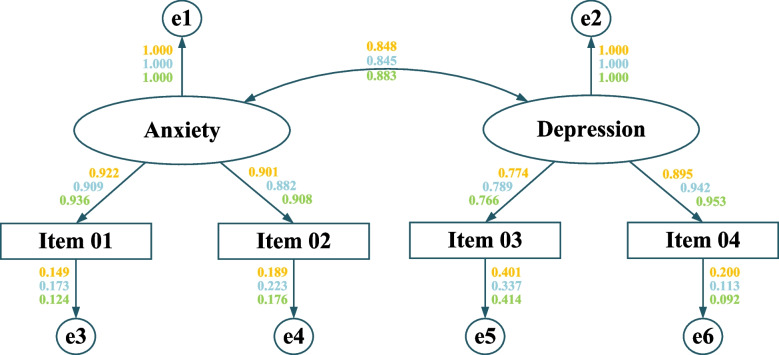
Confirmatory factor analysis results of the PHQ-4 for a two-factor model The one-sided arrows represent factor loadings while the double-sided one represents the covariance between the two factors. The orange, blue, and green color represent values of time 1, 2, and 3 respectively

As expected, the two-factor model of the PHQ-4-SC, as illustrated by CLI, TLI, and RMSEA, outperformed the other tested models (Table 1). All GOF indices showed that both the one-factor (CFI = 0.988–0.993; TLI = 0.965–0.978; RMSEA = 0.168–0.195) and the second-order (CFI = 0.926–0.943; TLI = 0.779–0.828; RMSEA = 0.404–0.534) models did not fit as well as the two-factor model (CFI = 1.000; TLI = 0.998–1.002; RMSEA = 0.000–0.056). Consequently, the two-factor model was selected for further evaluation of the measurement properties of the PHQ-4-SC (Fig. 1).

**Table 1:** Fit indices of different factor models of the PHQ-4

**Model**	***χ***^***2***^	***df***	**CFI**	**TLI**	**RMSEA (90% CI)**
Time 1					
One-factor Model	40.817	2	0.989	0.966	0.195 (0.146, 0.249)
** Two-factor Model**	**2.354**	**1**	**1.000**	**0.998**	**0.051 (0.000, 0.141)**
Second-order factor Model	200.622	2	0.943	0.828	0.441 (0.390, 0.493)
Time 2					
One-factor Model	34.73	2	0.988	0.965	0.179 (0.130, 0.233)
** Two-factor Model**	**0.004**	**1**	**1.000**	**1.002**	**0.000 (0.000, 0.014)**
Second-order factor Model	168.667	2	0.940	0.820	0.404 (0.354, 0.457)
Time 3					
One-factor Model	30.869	2	0.993	0.978	0.168 (0.119, 0.223)
** Two-factor Model**	**2.582**	**1**	**1.000**	**0.998**	**0.056 (0.000, 0.144)**
Second-order factor Model	292.886	2	0.926	0.779	0.534 (0.483, 0.586)
Threshold			≥ 0.900	≥ 0.900	≤ 0.080

Bold font means that this is the best-fit model

*Abbreviations*: *χ*^*2*^ Chi-square, *df* degrees of freedom, *CFI* comparative fit index, *TLI* Tucker-Lewis index, *RMSEA* root mean square error of approximation, *CI* confidence interval, *Δ* a change in *χ*^*2*^, *df*, CFI, TLI, and RMSEA

### Longitudinal measurement invariance

On the basis of the chosen two-factor model, we conducted the longitudinal CFA to test the measurement invariance of the PHQ-4-SC across time points. Using the GOF as indicators, the LMI analysis showed that all five models are fully supported as all values (CFI = 0.998–1.000, TLI = 0.998–0.999, and RMSEA = 0.017–0.024) and their changes (|ΔCFI|= 0.000–0.001, |ΔTLI|= 0.000–0.001, and |ΔRMSEA|= 0.000–0.007) fall within the cut-offs and remain in an excellent range (Table 2).

**Table 2:**
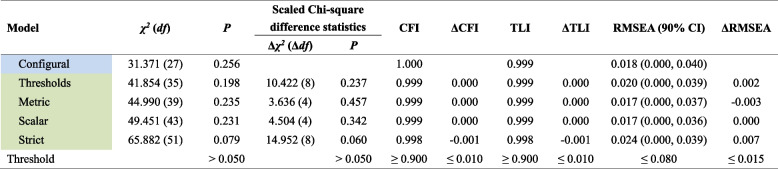
Fit indices of longitudinal measurement invariance of the PHQ-4 across three time points

Table shadings of the first column represent various meanings: 1) Blue represents that this is the configural model; 2) Green represents that this model is fully supported

*Abbreviations*: *χ*^*2*^ Chi-square, *df* degrees of freedom, *CFI* comparative fit index, *TLI* Tucker-Lewis index, *RMSEA* root mean square error of approximation, *CI* confidence interval, *Δ* a change in *χ*^*2*^, df, CFI, TLI, and RMSEA

### Construct validity

**Fig. 2:**
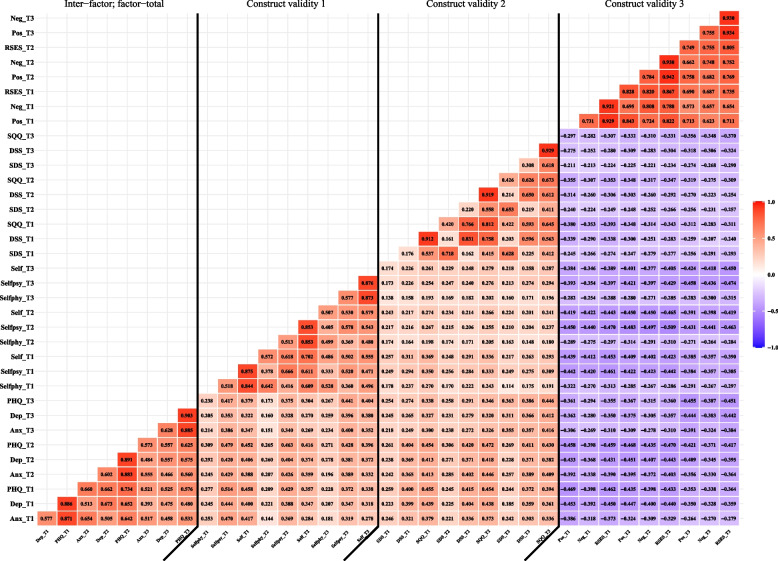
Inter-factor, factor-total, concurrent, and construct correlations between the PHQ, SQQ, and RSES across three time points Color gradient represents correlation level. Red represents a positive correlation. Purple represents a negative correlation Abbreviations: *Anx* anxiety, *Dep* depression, *PHQ* Patient Health Questionnaire, *Selfphy* self-rated physical condition, *Selfpsy* self-rated psychological condition, *Self* self-rated health condition, *SDS* sleep difficulty subscale, *DSS* daytime sleepiness subscale, *SQQ* Sleep Quality Questionnaire, *Neg* negative subscale, *Pos* positive subscale, *RSES* Rosenberg Self‑Esteem Scale, *T1* Time 1, *T2* Time 2, *T3* Time 3

Figure 2 shows the inter-factor, factor-total, and construct validity correlations of the PHQ-4-SC. We found moderate to high inter−factor and factor−total correlations with values ranging from 0.393–0.903. Most of the correlations of the PHQ-4-SC and its subscales with other measures were higher than 0.300. This partially supports the three hypotheses focused on the construct validity of the PHQ-4-SC.

### Internal consistency and test–retest reliability

We observed a good internal consistency of the PHQ-4-SC with Cronbach’s alpha values ranging from 0.818 to 0.919 and McDonald’s omega values ranging from 0.895 to 0.916 for baseline and follow-up. Similarly, most ICC values showed moderate to good test–retest reliability was shown by ICCs ranging from 0.505 to 0.717. Notably, only the ICC of the depression subscale across baseline and 15-week follow-up was outside the moderate range (ICC = 0.453). Detailed information on the internal consistency and test–retest reliability is provided in Table 3.

**Table 3:** Internal consistency and test–retest reliability of the PHQ-4

**Variables**	**PHQ**			**SQQ**			**RSES**		
	**Global**	**Anx**	**Dep**	**Global**	**SDS**	**DSS**	**Global**	**Pos**	**Neg**
**Cronbach’s α (95% CI)**									
T1	0.896 (0.881, 0.911)	0.907	0.818	0.809 (0.784, 0.834)	0.643 (0.592, 0.693)	0.835 (0.813, 0.857)	0.912 (0.901, 0.924)	0.866 (0.847, 0.885)	0.809 (0.783, 0.835)
T2	0.901 (0.886, 0.915)	0.890	0.853	0.845 (0.825, 0.866)	0.688 (0.644, 0.733)	0.872 (0.855, 0.889)	0.941 (0.934, 0.949)	0.910 (0.897, 0.923)	0.854 (0.834, 0.874)
T3	0.915 (0.903, 0.927)	0.919	0.844	0.858 (0.84, 0.877)	0.721 (0.681, 0.761)	0.866 (0.847, 0.884)	0.929 (0.920, 0.938)	0.901 (0.886, 0.915)	0.830 (0.806, 0.853)
**McDonald’s ω (95% CI)**									
T1	0.895 (0.881, 0.910)	-	-	0.807 (0.782, 0.832)	0.676 (0.633, 0.719)	0.839 (0.817, 0.860)	0.916 (0.905, 0.927)	0.863 (0.844, 0.882)	0.826 (0.802, 0.849)
T2	0.901 (0.887, 0.915)	-	-	0.845 (0.825, 0.865)	0.710 (0.671, 0.750)	0.875 (0.859, 0.892)	0.944 (0.937, 0.951)	0.904 (0.890, 0.917)	0.867 (0.849, 0.886)
T3	0.916 (0.904, 0.928)	-	-	0.857 (0.839, 0.876)	0.730 (0.693, 0.768)	0.869 (0.851, 0.886)	0.933 (0.924, 0.942)	0.893 (0.879, 0.908)	0.852 (0.832, 0.873)
**ICC (95% CI)**									
T1-T2	0.717 (0.672, 0.757)	0.646 (0.592, 0.694)	0.664 (0.612, 0.71)	0.811 (0.779, 0.839)	0.741 (0.696, 0.78)	0.828 (0.798, 0.853)	0.870 (0.839, 0.894)	0.836 (0.791, 0.870)	0.821 (0.790, 0.848)
T2-T3	0.622 (0.566, 0.673)	0.540 (0.476, 0.599)	0.556 (0.494, 0.613)	0.668 (0.616, 0.714)	0.669 (0.618, 0.714)	0.637 (0.581, 0.688)	0.818 (0.784, 0.847)	0.779 (0.741, 0.812)	0.777 (0.738, 0.810)
T1-T3	0.528 (0.462, 0.589)	0.505 (0.437, 0.568)	0.453 (0.382, 0.520)	0.637 (0.582, 0.686)	0.630 (0.572, 0.682)	0.576 (0.51, 0.635)	0.738 (0.661, 0.795)	0.711 (0.617, 0.778)	0.672 (0.614, 0.722)
**SEM**									
T1-T2	1.270	1.292	1.485	2.588	3.446	3.791	1.616	1.932	2.250
T2-T3	0.791	0.817	0.820	1.231	1.336	1.418	0.969	1.124	1.268
T1-T3	0.748	0.743	0.873	2.069	2.999	3.326	1.003	1.098	1.303

This table shows ordinal forms of Cronbach’s alpha (α) and McDonald’s omega (ω). Standard error of measurement was calculated as “SD × sqrt (1-ICC)”. The McDonald’s ω and the 95% confidential interval of Cronbach’s α cannot be calculated due to the anxiety and depression subscales containing only 2 items

*Abbreviations*: *PHQ* Patient Health Questionnaire, *Anx* anxiety subscale, *Dep* depression subscale, *SQQ* Sleep Quality Questionnaire, *SDS* sleep difficulty subscale, *DSS* daytime sleepiness subscale, *RSES* Rosenberg Self-Esteem Scale, *Pos* positive subscale, *Neg* negative subscale, *ICC* intraclass correlation coefficient, *CI* confidence interval, *SEM* standard error of measurement, *T1* Time 1, *T2* Time 2, *T3* Time 3

## Discussion

### Overall findings

Evidence of the measurement properties of the PHQ-4-SC from the current study revealed satisfactory performance in terms of structural validity, construct validity, and internal consistency, and of great importance, longitudinal measurement invariance over time. These results demonstrated that the PHQ-4-SC is a valid, reliable, and stable measure of depression and anxiety in the sample of health students.

### Structural validity

An identical two-factor structure of the PHQ-4-SC was observed, which is consistent with the original design of the PHQ and with other adaptations of the PHQ-4 [26, 27, 39]. The two subscales made adequate overall contributions to the PHQ-4-SC and have demonstrated the potential for it to support a bifactor model [92–94]. Therefore, screening for both depression and anxiety as a combined disorder, rather than either one or the other alone, is also advisable if the bifactor structure is identified in the future [26, 27, 39, 50].

### Longitudinal measurement invariance

The LMI, which was the core gap of the PHQ-4 in the Chinese culture, was fully supported and provided the first evidence for the longitudinal application of the PHQ-4 in China. Given that depression and anxiety covary across time points, our design of 1-week and 15-week intervals reveals the possible ability of the PHQ-4-SC to be used for both short terms and long periods [49, 50, 95]. However, it remains unknown whether its cross-sectional measurement invariance (CMI, e.g., gender) could be supported [49]. Further analysis of the CMI on the PHQ-4-SC, which is just as important as the LMI, is needed to complete the whole picture of assessing measurement invariance.

### Construct validity

Construct validity was suggested by the results of correlations between the PHQ-4-SC and the other three measures: the SRHQ, SQQ, and RSES. These are analogous to some other international studies and point to the special characteristics of depression and anxiety—as a signal for psychosomatic disorders [39]. However, a missing part of the construct validity is the lack of correlations with instruments measuring similar constructs (e.g., Center for Epidemiologic Studies Depression Scale). Completing the missing part of the study would be preferable to fill the gap in assessing the construct validity of the PHQ-4 applied in the Chinese population.

### Internal consistency and test–retest reliability

Despite this ultra-short instrument consisting of only four items (two items for both subscales), the internal consistency was more robust than we expected [27]. This may be due to the face validity of the items, which made them easy to understand in Chinese [49, 64]. As for the only non-ideal ICC value, we speculate that this may be due to the long-term interval of 15 weeks—this could reduce the status of the healthcare students when they repeatedly answer the same questionnaire. This phenomenon has also been observed in another similar study [96].

### Strengths and limitations

Several strengths should be highlighted. First, to date, this is the first study to evaluate the LMI of the PHQ-4-SC in a sample of the Chinese population, and to assess its use across time points. Second, this is the first study to examine the measurement properties of the PHQ-4-SC with a design including both a short-term and a long-term interval. Last, the study used multiple instruments to examine its construct validity, thus providing initial data for the analysis of risk factors for mental disorders.

Nevertheless, the study also had several limitations. First, we did not assess cross-sectional measurement invariance. Future comparisons between subgroups or characteristics should be made with caution. Second, the bifactor model was not subsequently assessed to confirm the unidimensional properties of the PHQ-4-SC. Further testing of this model would be promising for the proficiency of the overall validity of the PHQ-4-SC. Last, construct validity is the lack of correlations with instruments measuring similar constructs. Researchers are more than welcome to concurrently use other similar instruments to measure depression and anxiety simultaneously.

## Conclusion

The factor structure, longitudinal measurement invariance, construct validity, internal consistency, and test–retest reliability of the Chinese version of the PHQ-4 were demonstrated across three waves of measurement. Such evidence lends support for the continued and expanded use of the PHQ-4 as an effective screening instrument in China.

### Supplementary Information

Supplementary Material.

## Data Availability

The datasets analyzed during the current study are not publicly available due to the personal health information of participants needing to be protected but are available (de-identified data) from the corresponding author on reasonable request.

## Abbreviations

CFA: confirmatory factor analysis
CFI: comparative fit index
CMI: cross-sectional measurement invariance
COSMIN: COnsensus-based Standards for the selection of health Measurement INstruments
GAD-2: two-item Generalized Anxiety Disorder
GOF: goodness-of-fit
ICC: intraclass correlation coefficient
LMI: longitudinal measurement invariance
PHQ-2: two-item Patient Health Questionnaire
PHQ-4: four-item Patient Health Questionnaire
RMSEA: root mean square error of approximation
RSES: Rosenberg Self-Esteem Scale
SQQ: Sleep Quality Questionnaire
SRHQ: Self-Rated Health Questionnaire
TLI: Tucker-Lewis index
WLSMV: weighted least squares mean and variance adjusted
