# Supplementary material for: Longitudinal measurement invariance and psychometric properties of the Patient Health Questionnaire-Four in China
Source: BMC Psychiatry. 2024 Jul 22;24:517. doi: 10.1186/s12888-024-05873-2 (PMC11265176; doi:10.1186/s12888-024-05873-2)
Supplement: Supplementary file 1 — Supplementary Material. [file 12888_2024_5873_MOESM1_ESM.docx]

**Supplementary Material**

**Table S1** The constrained parameters for various measurement models

| **Models** | **Thresholds** | **Factor loadings** | **Intercepts** | **Residuals** |
| --- | --- | --- | --- | --- |
| Configural |  |  |  |  |
| Thresholds | ⚪ |  |  |  |
| Metric | ⚪ | ⚪ |  |  |
| Scalar | ⚪ | ⚪ | ⚪ |  |
| Strict | ⚪ | ⚪ | ⚪ | ⚪ |

⚪ constrained parameter

**Table S2** Demographic characteristics of the participants (*N* = 512)

| **Variable** | **n (%)** | **PHQ mean scores (SD)** | | |
| --- | --- | --- | --- | --- |
|  |  | **Time 1** | **Time 2** | **Time 3** |
| **Gender** | | | | |
| Male | 118 (23.047) | 3.203 (2.202) | 3.093 (1.921) | 3.017 (2.188) |
| Female | 394 (76.953) | 3.881 (2.421) | 3.624 (2.139) | 3.495 (2.144) |
| **Age^*^** | | | | |
| < 20 | 155 (30.333) | 3.819 (2.508) | 3.645 (2.267) | 3.555 (2.222) |
| ≥ 20 | 356 (69.667) | 3.683 (2.338) | 3.441 (2.028) | 3.309 (2.136) |
| **Home location** | | | | |
| Urban | 185 (36.133) | 3.771 (2.421) | 3.341 (1.839) | 3.313 (2.170) |
| Rural | 196 (38.281) | 3.568 (2.260) | 3.528 (2.229) | 3.284 (2.101) |
| Suburban | 131 (25.586) | 3.990 (2.590) | 3.721 (2.227) | 3.731 (2.261) |
| **Only child** | | | | |
| Yes | 205 (40.039) | 3.620 (2.329) | 3.332 (2.003) | 3.308 (2.248) |
| No | 307 (59.961) | 3.796 (2.427) | 3.618 (2.162) | 3.438 (2.102) |
| **Monthly households' income** | | | | |
| < 10,000 | 222 (43.359) | 3.590 (2.267) | 3.414 (2.125) | 3.338 (2.105) |
| ≥ 10,000 | 290 (56.641) | 3.828 (2.474) | 3.569 (2.084) | 3.421 (2.206) |
| **Part-time status** | | | | |
| Do part-time job | 88 (17.188) | 3.582 (2.372) | 3.224 (2.013) | 3.357 (2.087) |
| No part-time job | 424 (82.812) | 3.758 (2.392) | 3.568 (2.119) | 3.391 (2.181) |
| **Leisure time sports involvement** | | | | |
| Yes | 251 (49.023) | 3.745 (2.489) | 3.573 (2.136) | 3.427 (2.167) |
| No | 261 (50.977) | 3.691 (2.211) | 3.382 (2.041) | 3.314 (2.156) |
| **Engagement in hobbies** | | | | |
| Yes | 359 (70.117) | 3.656 (2.343) | 3.389 (2.088) | 3.341 (2.145) |
| No | 153 (29.883) | 3.875 (2.482) | 3.750 (2.116) | 3.481 (2.201) |
| **Preferred coping strategies** | | | | |
| Active coping | 303 (59.180) | 3.911 (2.391) | 3.619 (2.157) | 3.436 (2.151) |
| Push through | 167 (32.617) | 3.203 (2.113) | 3.064 (1.888) | 3.080 (1.917) |
| Ignore problems | 42 (8.203) | 5.000 (3.055) | 4.912 (2.036) | 4.618 (2.975) |

* The Age group contains 1 missing value

Abbreviations: *PHQ* Patient Health Questionnaire, *SD* standard deviation


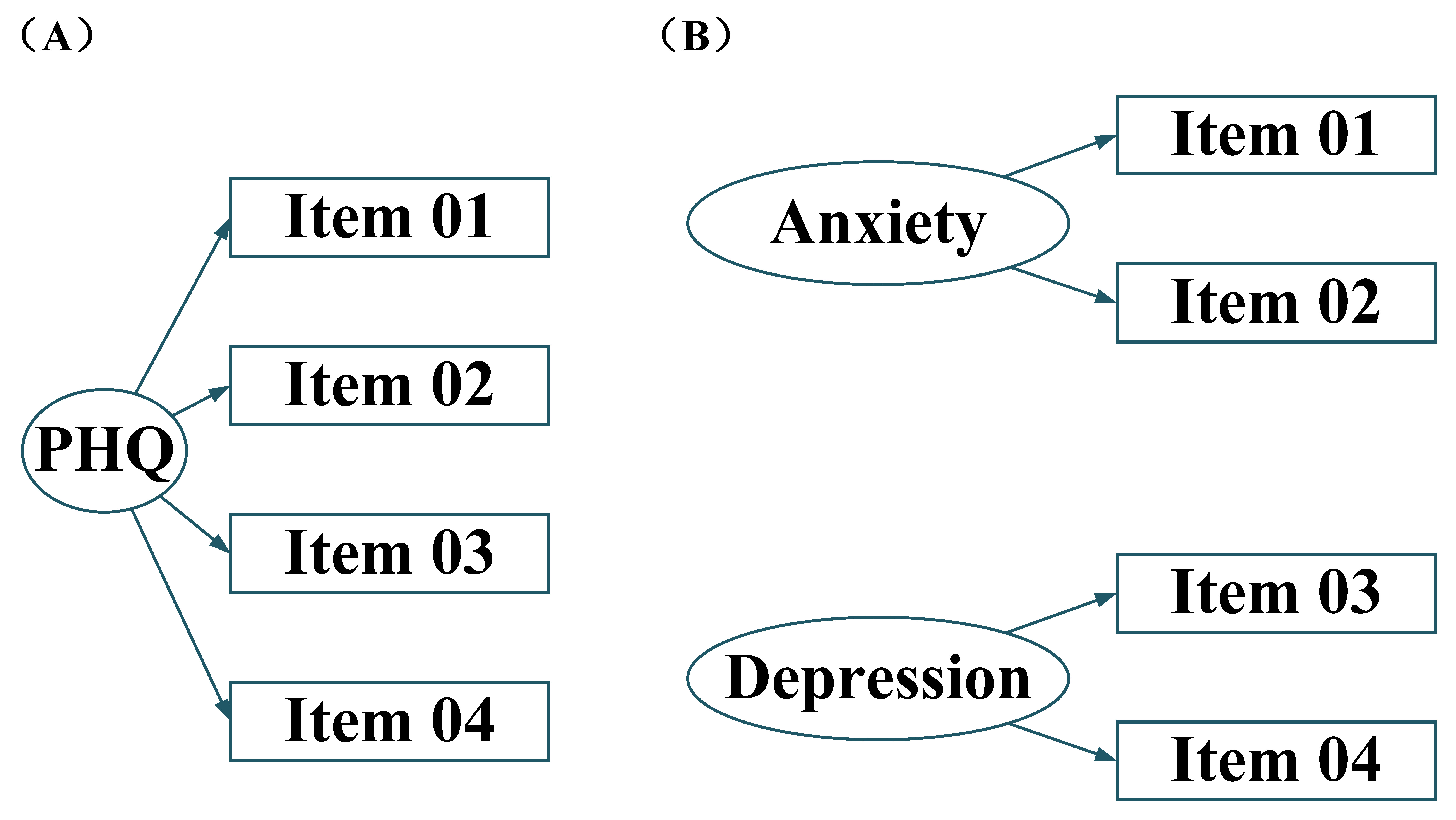


**Figure S1** Illustration of optional structures for the PHQ-4: (A) One-factor structure and (B) Two-factor structure

Abbreviations: *PHQ* Patient Health Questionnaire
